# The First World Congress on Tourette Syndrome and Tic Disorders: Controversies and Hot Topics in Etiology and Treatment

**DOI:** 10.3389/fnins.2016.00246

**Published:** 2016-06-07

**Authors:** Carol A. Mathews, Jeremy S. Stern

**Affiliations:** ^1^Department of Psychiatry, College of Medicine, University of FloridaGainesville, Florida, USA; ^2^Department of Neurology, St George's University of LondonLondon, UK

**Keywords:** Tourette, tics, deep brain stimulation, dopamine, GWAS

## Abstract

The first World Congress on Tourette Syndrome and Tic Disorders was held in London, June 2016 by the Tourette Association of America, Tourettes Action (UK), and the European Society for the Study of Tourette Syndrome. Presentations arising from large-scale collaborative projects were an important component of the scientific programme. This article focuses on areas raised in the hot topics session and two moderated debates, which covered emerging research in etiology and treatment. The hot topics ranged across genetics, arguably including the first confirmed Tourette Syndrome (TS) susceptibility gene *NRXN1*, neurocognition, and neurophysiology, including the possibility of a neurocognitive endophenotype for TS and the use of depth and cortical surface electrodes to investigate the neurophysiology of tics on the background of the evolving field of deep brain stimulation (DBS), to novel treatment approaches such as dental orthotics and an online behavioral intervention. The debates aired controversies in treatment; pharmacotherapy vs. behavioral treatment and the place of medical cannabinoids. These sessions demonstrate the vibrancy of a field that has considerably expanded in the last decade, the significant progress that has been made, and the direction that some of the most fruitful next phases of research will take.

## Introduction

These sessions of the congress were devoted to late-breaking studies and hot topics, including controversies in the field of Tourette Syndrome (TS) research or treatment. The presentations fell into two main themes, the first, elucidating the etiology of TS, and the second, the identification of novel or controversial treatments for TS. These presentations highlight the importance of large-scale collaborative efforts in the study of TS and provide evidence that, after many years of incremental advances, with collaborative efforts more substantial discoveries may be just around the corner. This is best illustrated in the genetic studies, where nearly 100 clinicians and scientists contributed clinical samples and expertise, and in the studies of environmental risk factors, which took place using the Avon Longitudinal Study of Parents and Children (ALSPAC; Golding et al., 2001), a birth cohort in which data has been collected, curated, and studied for over 20 years by hundreds of researchers. Such large scale, collaborative efforts are also becoming the norm for studies examining the efficacy and safety of TS treatments, whether in the form of meta-analyses of multiple small investigator-initiated studies, or in the form of large, multi-institution investigations of a specific treatment.

TS has long been known to be a complex disorder etiologically, with both genetic and non-genetic contributors. However, clear specific risk factors for TS, either genetic or environmental, have been difficult to identify and/or replicate. The availability of large samples of individuals with extensive phenotype and/or genotype data, some population-based, and some clinically ascertained, have recently led to advances in our understanding of the causes of TS. Although, TS is one of the most heritable of the neurodevelopmental disorders (Pauls et al., 2014b), with heritability estimates of 60–80% (Davis et al., 2013), the last 30 years of genetic studies, including recent genome-wide association studies, have been inconclusive. These studies indicate that TS is highly polygenic; that is, hundreds (or perhaps thousands) of genes of small effect contribute to TS risk in an additive manner. For this reason, tens of thousands of samples will likely be needed to identify individual TS susceptibility variants using genome-wide approaches. However, the currently available sample sizes, while falling short of what is needed for comprehensive identification of the genes and gene variants responsible for TS, may be efficiently used for gene discovery using alternative approaches.

### Genetic studies

Two presentations in this session focused on such alternative approaches to dissecting the genetic etiology of TS, and demonstrate the value of complementary scientific approaches. In the first, Alden Huang (University of California, Los Angeles), working with the Tourette Syndrome Association International Consortium for Genetics (TSAICG), examined the relationship between TS and copy number variants (CNVs) in 2764 individuals with TS and 2853 ethnically matched controls. Analyses were limited to large (>400 kilobases), rare (< 1% prevalence) CNVs, which are likely to be pathogenic. Huang identified multiple recurrent CNVs in genomic regions that have been previously implicated for TS, as well as substantial overlap with CNV regions that have been implicated in other neurodevelopmental disorders, including autism spectrum disorders (ASD) and intellectual disability(Grayton et al., 2012). Two of these regions showed an enrichment of CNVs in TS cases compared to controls. These were *NRXN1* (1-sided Fisher's exact, *p* = 0.007), which has been previously reported to be associated with TS and *CNTN4* (*p* = 0.029). All of the CNVs detected in *NRXN1* were deletions, consistent with the prior literature (Nag et al., 2013; McGrath et al., 2014), while both deletions and duplications were present in the *CNTN4* locus. At the time of the Congress *NRXN1* may be considered the first confirmed susceptibility gene for TS.

The second study used the same dataset to conduct gene pathway analyses. There are many forms of pathway analyses, but the basic idea is to identify enrichment of genetic variants within specific known gene pathways or gene sets. Like the CNV analyses discussed above, an advantage of pathway analyses is that they can be effective in relatively small sample sizes, typically requiring thousands rather than tens of thousands of samples. This work was conducted by Fotis Tsetsos (University of Thrace), in conjunction with the TSAICG. Tsetsos used two complementary statistical approaches to examine relationships between TS and gene pathways defined from multiple sources, including curated gene sets from the published literature, computational gene sets defined from cancer-oriented microarray data, genes annotated using the same GO search terms, genes that share a microRNA binding motif, etc. Variants associated with nervous system tissues, in particular, parietal cortex and basal ganglia, were enriched in these analyses. A gene set with promotor regions around TCF3 (transcription factor 3) was also implicated in TS etiology (corrected *p* = 0.006). TCF3 is a member of the HLH (helix-loop-helix) family of transcription factors, and is thought to regulate developmental patterning processes in the central nervous system. TCF3 also suppresses Wnt, a protein that is involved in neuronal differentiation and proliferation of neural development cells (Gribble et al., 2009).

### Studies of non-genetic risk factors

Genetic causation accounts for ~60% of TS risk, suggesting that other, non-genetic (environmental) factors are also very important in the development of this disorder. Previous work in both clinical and population-based samples have implicated a number of pre- and perinatal risk factors for TS, including prenatal maternal smoking, prenatal maternal alcohol use, and possibly maternal parity and weight gain during pregnancy (Mathews et al., 2006, 2014; Pringsheim et al., 2009; Motlagh et al., 2010). In the third study in this session to focus on the etiology of TS, Yoav Ben-Shlomo (University of Bristol), and his colleagues used the ALSPAC sample to examine another type of potential environmental risk factor for TS, maternal anxiety and depression during pregnancy. The ALSPAC cohort is a prospective pre-birth cohort that has followed children born in Avon, UK in 1992 and their parents for over 20 years, and has collected extensive phenotypic data (Golding et al., 2001). Ben-Shlomo compared self-reported anxiety and depressive symptoms for both mothers and fathers at four time points, two prenatal (18 and 32 weeks), and two postnatal (18 weeks and 8 months after delivery) for children with chronic tic disorders including TS (TS/CT) and a control sample of children without chronic tics (Ben-Shlomo et al., 2016). Socioeconomic measures and other relevant potential confounders were controlled for in the analysis. After correction for potential confounders, chronic maternal anxiety (present both pre- and post-birth) and pre-natal maternal depression (but not post-natal maternal depression) were significantly associated with TS/CT (odds ratio = 2.17, *p* = 0.007; odds ratio 1.86, *p* = 0.04, respectively). Paternal anxiety and depression were not significantly associated with TS/CT. These findings suggest that maternal psychopathology may be a risk factor for TS and other chronic tic disorders. Maternal chronic anxiety may in fact represent a shared genetic susceptibility for TS, as this variable was associated with TS/CT both pre-and post-natally. In contrast, maternal depression may represent a time-specific environmental risk factor for TS, perhaps representing medication use during pregnancy, or intra-uterine neuroendocrine effects of stress. It should be noted, however, that both associations require confirmation in independent datasets.

The final study pertaining to the etiology of TS in this session was a systematic review focused on neurocognitive performance in individuals with TS. This study contributes to a growing literature on potential endophenotypes for TS and other complex disorders. An endophenotype is a heritable, measurable trait or feature that is associated with a disorder of interest, but is state independent (e.g., manifests in individuals whether or not they are manifesting the disorder, including in unaffected family members). No endophenotypes have yet been identified for TS, but specific neurocognitive abnormalities have been suggested as potential endophenotypes for two related disorders that are highly comorbid with TS, obsessive compulsive disorder (OCD; Pauls et al., 2014a), and attention deficit hyperactivity disorder (ADHD; Pineda et al., 2011; Eddy and Cavanna, 2014; Peskin et al., 2015). The study by Beth Hobson (University of Birmingham), and her colleagues, takes the first step in identifying potential TS endophenotypes by investigating whether neurocognitive dysfunction is consistently associated with TS. A search of PubMed, Medline, and PsychINFO identified 12 relevant studies, four of which included children and/or adolescents. In general no consistent differences in neurocognitive function between TS cases and controls were found. The one possible exception was in the area of cognitive inhibitory control. Individuals with TS showed a trend toward verbal inhibitory deficits, although this finding did not reach the level of statistical significance. Inhibitory control in TS, typically motor inhibition, but also cognitive inhibition, may lie at the heart of the neurology of TS and is an active area of investigation requiring future study.

## Treatment

Management of TS is challenging and has remained largely unsatisfactory through the last decade of intensifying clinical and scientific interest in the condition. In clinical terms, given the spectrum nature of the presentation, it is important to define the treatment target in each case, as comorbidities such as ADHD or OCD are commonly more impairing than are the tics themselves. Tics often improve over the course of adolescence and at present their treatment is overall less reliable and less evidence based than treatments for the commonly co-occurring disorders. However, tics can be extremely severe in up to 15% of cases, and their effect on functioning varies greatly between individuals. Where tics are severe or intrusive, pharmacotherapy can be considered. The index drug was haloperidol in the 1950s, and since then a variety of neuroleptics have been used, including newer or atypical agents (Hartmann and Worbe, 2013). The dopamine hypothesis as a substrate for TS essentially originated from this clinical association and has been variably substantiated in more recent functional imaging and other work (Singer et al., 1982; Segura and Strafella, 2013). An alpha-2 adrenergic agonist, clonidine, is well-established and other classes of drugs with some support for efficacy in TS treatment include the anticonvulsant Topiramate and the dopamine depleter tetrabenazine. Treatment efficacy for each option is variable. There is relatively little randomized controlled data, sparse head-to-head comparisons, and the available Class 1 evidence needs to be considered in the context of generally short-term trials conducted over the course of only weeks in a condition that is hard to objectively measure and naturally fluctuates, whereas in clinical practice an initial positive response with less benefit over time is commonly seen. There are several reviews and recommendations for drug treatment and the first truly systematic review and meta-analysis is in press (Roessner et al., 2011; Hollis et al., 2016).

The other conventional modality of treatment is behavioral. These have evolved from the early exploratory literature into evidence-based schedules based around strategies either designed to suppress tics by using competing responses to premonitory urges that precede tics (e.g., Habit Reversal Training; HRT) or to increase tolerance of the premonitory urges (e.g., Exposure with Response Prevention; ERP). HRT has been incorporated into a package called Comprehensive Behavioral Intervention for Tics (CBIT) which was effective in both children and adults in two influential randomized trials of 10 weeks therapy followed up for 6 months (Wilhelm et al., 2012).

In addition to the conventional treatments of pharmacotherapy and behavioral therapy, alternative approaches are also evolving, ranging from neurosurgical stereotactic deep brain stimulation (DBS), which has some evidence base, although not yet extensive, and other more controversial possibilities, such as oral orthotic devices and the use of medical cannabinoids. The treatment talks in this session focused on (1) the more controversial approaches to treating tics and (2) alternative approaches to delivering the more conventional treatments.

John Walkup (Cornell Weill Medical Center), presented the methodology and preliminary results from a TSA sponsored study of an oral orthotic device (an occlusal splint). This treatment evolved out of observations from the dental community that dental orthotics reduce tics anecdotally, with an underlying hypothesis that TS is caused by a brainstem response to dental factors rather than being a genetic neurodevelopmental syndrome (Sims and Stack, 2009). This hypothesis and corresponding treatment approach did not gain initial traction amongst neuropsychiatrists. However, patients and parents in a number of countries have been willing to try occlusal splints, sometimes at significant expense, leading to a real need for a high quality clinical trial. Walkup presented a double blind placebo controlled randomized study using the occlusal splint compared to sham orthotics over 2 weeks, with assessment of durability of effect over a further 4–6 weeks. Outcome measures include changes in tic severity, improvement in functioning, and assessments of acceptability and patient satisfaction. To date, open-label pilot studies of the intervention have found it to be feasible, acceptable and non-harmful. The first three participants had high satisfaction despite mild to moderate adverse effects (sore mouth, excess salivation etc.) and had reduced tic severity with two participants being very much improved on measures of functioning for the initial 2 weeks, although benefit was not sustained at this level for the remaining 4–6 weeks.

Michael Himle (University of Utah), presented the development of “TicHelper,” a self-administered online tool for teaching or delivering CBIT from the team that have developed the treatment. If this mode of delivery is successful, there would be immediate potential impact on clinical practice, as specialist psychology resources are limited in most countries, particularly in non-urban areas, so that behavioral therapy often cannot be delivered. In a pilot study, the investigators selected 8 children to use the program for 2 weeks to target a single tic. 7/8 children showed a much increased awareness of tics and were able to demonstrate appropriate use of a competing response, all important components of successful CBIT. Longer term outcomes, including durable improvement of tics, are not yet available.

As noted previously, efforts are underway to understand the neurophysiology and etiology of TS, but much more work is yet to be done. Understanding the neurophysiology of the generation and control of tics and their neural correlates is relevant to identifying and refining appropriate treatments for this complex disorder. This is well-illustrated in the continuing questions over the most effective surgical target for DBS, the most radical of the existing treatments for TS, and the selection of patients likely to benefit. Non-invasive data mapping the neurological substrate for TS is available from functional radioisotope and magnetic resonance imaging and at an altogether different temporal and anatomical resolution by recording from DBS electrodes (Bour et al., 2015). On behalf of Shute et al., Aysegul Gunduz (University of Florida) presented a unique study examining two patients who were implanted with both subdural electrodes (primary motor, M1, and premotor, PM cortices) and depth electrodes (thalamic centromedian nucleus, Cm). Awake recordings were made of local field potentials (thalamus) and electrocorticograms (cortex) with the patient ticcing, suppressing tics, making voluntary movements, and imitating tics. Regionally specific activation patterns were suggested by phase amplitude coupling analysis (PAC). A dissociation was found between ticcing in which contralateral low frequency activity in all three areas was seen and for voluntary movements in which only the cortex was active. In one patient, tics could be detected electrophysiologically using this approach with 70% sensitivity and specificity. This complements the better established field of PAC changes in Parkinson's disease and its treatment with DBS and also opens further possibilities for capture and treatment of tics within closed loop adaptive DBS systems (Almeida et al., 2015).

### Controversies in treatment

In addition to the scientific presentations, the two congress debate sessions focused on treatment, and in particular, on controversies in treatment. The first explored CBIT vs. pharmacotherapy as first line treatment, and was chaired by Stanley Fahn (Columbia University), and presented by Douglas Woods (Texas A & M University; advocating CBIT) and Donald Gilbert (Cincinnati Children's Medical Center; advocating pharmacotherapy). Like all good conference debates, fair amounts of devil's advocacy and inventiveness were employed, reflecting the underlying truths that all clinicians are grappling with- drugs are not as reliably effective as we would like and commonly cause adverse effects (usually mild), CBIT and other behavioral interventions are not as accessible as they should be due to lack of funding and experienced practitioners within local reach. An important point that was raised during the debate was the fact that there are no comparative studies of the two modalities of treatment, and that such studies are necessary.

The other topic was the use of cannabinoids (including marijuana) in the treatment of TS, and was chaired by Joseph Jankovic (Baylor College of Medicine) and energetically debated by Kirsten Mueller-Vahl (Hannover School of Medicine; representing the pro-cannabinoid stance), and Paul Sandor (University of Toronto; representing the anti-cannabinoid stance). The underlying hypothesis was that neurotransmitters other than dopamine, including endocannabinoids, are likely to be important substrates of various aspects of TS. Endocannabinoids are thought to modulate many other classes of neurotransmitter, including monoamines with a high density of CB1 receptors in the basal ganglia. There are limited case reports and two controlled trials of Delta 9-tetrahydrocannabinol (THC; Muller-Vahl et al., 2002, 2003) which followed single dose studies. However, a comprehensive Cochrane review concluded there is insufficient evidence for clinical use (Curtis et al., 2009). As with the other controversial treatments, this area is worthy of further study. In clinical practice it is uncommon for adults in the UK to self-medicate with marijuana despite fairly frequent recreational use, which is in contrast to a German interview study (Muller-Vahl et al., 1997). Use of the medically isolated component of THC may offer different or more reliable effects, perhaps within the usual context of drug treatment of TS in which efficacy of all evidence-based options varies between individuals.

## Conclusions and future directions

As can be seen in the work presented in this session, research on the causes and treatment of TS is at a turning point. While much progress has been made in the last 10 years, there is still much to be done. In order to make substantial progress, collaboration is required, not only between investigators in similar fields, but also between scientists and clinicians across disciplines, and between scientists, clinicians, advocacy groups, and patients and families. Such collaborative efforts have been enormously successful in propelling forward breakthroughs in identifying genetic causes of ASD, new and novel treatments for cancer, to name two of many examples. Only with broad support and participation within and across constituencies, as well as a willingness to take risks, will we be able to make real strides forward toward a better understanding of this disorder, and toward effective identification and treatments.

## Author contributions

JS part drafted and edited the manuscript. CM part drafted and edited the manuscript. JS and CM co-chaired the hot topics session at which some of the work was presented.

### Conflict of interest statement

CM is co-chair of the TAA scientific advisory board and the co-chair of the first World Congress on TS and Tics scientific committee. He received travel funding to attend the World Congress from the TAA. JS is honorary medical director of Tourettes Action and was co-chair of the 1st World Congress on TS and Tics scientific committee.
